# CRSwNP-derived cells retain native disease-relevant characteristics in vitro

**DOI:** 10.1186/s12950-026-00497-7

**Published:** 2026-03-19

**Authors:** Philipp Kühnel, Luisa Vossler, Melanie Siemen, Holger Sudhoff, Ingo Todt, Karsten Niehaus, Matthias Schürmann

**Affiliations:** 1 Department of Otolaryngology, Head and Neck Surgery, Campus Klinikum Bielefeld Mitte, University Hospital OWL of Bielefeld University, Bielefeld, Germany; 2 Proteome and Metabolome Research, Center for Biotechnology (CeBiTec), Faculty of Biology, Bielefeld University, Bielefeld, Germany; 3 Kopfzentrum Bielefeld, Bielefeld, Germany; 4 Department of Otolaryngology, Alexianer St. Gertrauden-Krankenhaus, Berlin, Germany

**Keywords:** Chronic rhinosinusitis, Phenotypic in vitro model, Intrinsic cellular differences, Type 2 inflammation, Pathogen associated molecular pattern, Biologic

## Abstract

**Objective and design:**

Chronic rhinosinusitis (CRS) is a heterogeneous inflammatory disease of the paranasal sinuses, which is divided into CRS with nasal polyps (CRSwNP) and CRS without nasal polyps (CRSsNP). CRSwNP is typically caused by type 2 inflammation, which is characterized by elevated IL-4 and IL-13 levels, impairment of the epithelial barrier, and tissue remodeling. While the involvement of immune cells is well known, it remains unclear to what extent structural cells intrinsically maintain disease-specific functional programs. The aim of this study was to determine whether epithelial cells and fibroblasts derived from CRSwNP and CRSsNP differ in their barrier properties, inflammatory reactivity, and type 2-associated functional characteristics.

**Methods:**

Air–liquid interface (ALI) epithelial cultures and primary fibroblast cultures were generated from CRSwNP and CRSsNP tissue. Epithelial barrier integrity was assessed by transepithelial electrical resistance (TEER), and inflammatory responses to TLR stimulation were analyzed by qRT-PCR. Fibroblast migration was evaluated using scratch assays. Cellular responses to IL-4/IL-13 with or without Dupilumab were quantified by qRT-PCR.

**Results:**

CRSwNP-derived epithelial cells exhibited delayed tight junction formation and impaired differentiation compared to CRSsNP cells. Poly(I:C) stimulation induced stronger expression of Th2-associated cytokines in CRSwNP cultures. CRSwNP fibroblasts showed reduced migratory capacity and a heightened induction of Th2 cytokines and extracellular matrix genes following IL-4/IL-13 stimulation relative to CRSsNP fibroblasts.

**Conclusion:**

Epithelial cells and fibroblasts derived from CRSwNP retain disease-associated type 2 characteristics in vitro, indicating persistent disease-aligned programmed functional alterations of the polyp microenvironment. In contrast, CRSsNP-derived cells lacked comparable enhanced type 2 responsiveness. These findings support CRSwNP as a distinct, self-sustaining inflammatory endotype and underscore the value of patient-derived models for investigating disease mechanisms and targeted therapies.

**Supplementary Information:**

The online version contains supplementary material available at 10.1186/s12950-026-00497-7.

## Introduction

Chronic rhinosinusitis (CRS) is a common and often distressing condition characterized by persistent inflammation of the paranasal sinuses. Typical symptoms resemble the common cold such as persistent nasal obstruction, facial pain, reduced olfactory perception and nasal secretions but will persist over a period of more than 12 weeks. The etiology of CRS is complex and includes infectious, allergic and environmental factors. In addition, impaired immune regulation plays a decisive role in the pathogenesis of the disease [1, 2]. A key differentiation is based on the presence or absence of nasal polyps (NP), which defines two main phenotypes: CRS with NP (CRSwNP) and CRS without NP (CRSsNP). This classification is important not only for diagnosis but also for the choice of therapeutic strategies [3, 4].

The underlying chronic inflammation in CRS is mediated by a complex network of cytokines and chemokines. While CRSwNP is typically characterized by a type 2 inflammatory response with an increased expression of Interleukin-4 (IL-4), Interleukin-5 (IL-5) and Interlekin-13 (IL-13) and a strong eosinophil infiltration, CRSsNP is often associated to a non-eosinophilic inflammation dominated by neutrophil granulocytes and an increased production of pro-inflammatory cytokines such as interferon-gamma (IFN-γ), and tumor necrosis factor alpha (TNF-α) indicating a strong Th1-mediated inflammatory response [5, 6]. Impaired barrier function of the nasal mucosal epithelium is one of the fundamental pathophysiological changes in CRS. Studies have revealed that CRS patients exhibit insufficiently developed tight junctions accompanied by a significant reduction in transepithelial electrical resistance (TEER) [7–9] and increased apoptosis of epithelial cells compared to healthy individuals [10]. This reduced barrier increases susceptibility to e.g. microbial infections. The corresponding pathogen associated molecular pattern (PAMP) entering the mucosa directly accelerate inflammation via Toll like receptor (TLR) signaling in polyp tissue [11]. While epithelial cells can contribute to tissue inflammation through the secretion of thymic stromal lymphopoietin (TSLP), IL-25 and IL-33 following TLR signaling, it is also known that viral infections can induce the release of IL-8 and TNF-α from epithelial cells. IL-25, IL-33, and TSLP promote the differentiation and maturation of Th2 cells. This further increases the production and release of the key Th2 cytokines IL-4 and IL-13, which play a critical role in maintaining the type 2 inflammatory response [12–15].

Alongside epithelial cells, fibroblasts also play a decisive role in the epithelial remodeling of NP. They are able to promote airway remodeling e.g. by generating extracellular matrix (ECM) proteins [16, 17]. Fibroblasts excessively produce ECM proteins like collagens and fibronectin (FN1) in the inflammatory niche, which are believed to play a crucial role in polyp formation and persistence of this tissue alteration [18–20]. As sentinel cells they respond to pro-inflammatory signals such as IL-4 and IL-13 and enhance Th2 signaling by producing chemotactic molecules. It is known, that fibroblasts are able to secrete large amounts of eotaxins like CCL11, CCL26, and the matricellular protein periostin (POSTN). In airway derived fibroblasts CCL26 and POSTN are known to be upregulated by IL-4 and IL-13 [20–23] but CCL11 was only shown to be induced by TLR4 agonists [24]. CCL11 and CCL26 act as chemoattractants that regulate eosinophil infiltration, which in turn contributes to epithelial disruption via ECP release [25]. POSTN plays a crucial role in various allergic, inflammatory, and fibrotic diseases, with its involvement in airway disorders such as asthma, chronic obstructive pulmonary disease, allergic rhinitis, and idiopathic pulmonary fibrosis being well-documented [26, 27] Beyond its role in inflammation, POSTN further promotes tissue remodeling by enhancing eosinophil adhesion, supporting fibroblast activation, and stabilizing the extracellular matrix, thereby reinforcing the contribution of fibroblasts to the type 2 inflammatory environment [28, 29].

One promising therapeutic option to break through this type 2 inflammation is Dupilumab, a fully human monoclonal antibody that binds to IL-4Rα, effectively blocking the signaling of both IL-4 and IL-13. It has demonstrated efficacy in several Th2-mediated conditions, including asthma, atopic dermatitis, and CRSwNP. By modulating the key cytokines of type 2 inflammatory responses in CRSwNP patients, Dupilumab may help improve symptoms and reduce polyp formation, which is particularly useful in difficult-to-treat cases [28, 30].

CRSsNP and CRSwNP are characterized by opposing inflammatory signaling. Hence, the response to therapeutic approaches are distinguishable as well [31]. A direct comparison between these two manifestations would provide a deeper insight into their pathogenesis. Unfortunately, most published in vitro studies either exclusively investigate the pathogenesis of CRSwNP in comparison to healthy mucosa or considered all CRS patients as a homogeneous group without acknowledging the specific characteristics of the respective subtypes [32]. Hence, our goal was to investigate the differences in two cell culture models of respiratory epithelium grown at the airliquid-interface (ALI) and fibroblasts derived from nasal tissue of CRS patients. Focusing on the typical cellular properties like epithelial barrier function and underlying inflammatory mechanisms of polyp development described above.

## Materials and methods

### Tissue collection

For the cultivation of the respiratory epithelium and fibroblasts, tissue samples were taken from the nasal mucosa (NS) of patients with CRSsNP as well as NP (SP) of patients with CRSwNP. Specimens were obtained during endoscopic pansinus surgery after written informed consent was obtained from the patients according to local and international guidelines (Ethics Committee of the university hospital Ruhr-Universität Bochum in Bad Oeynhausen, Germany; 20108-397_4) at the University Department of Otorhinolaryngology, Head and Neck Surgery, Bielefeld. The specimens were clearly classified as inflamed NS or polyp tissue based on a histopathological examination, confirmed in the pathology report. In addition, tissue eosinophilia was histologically evaluated by the pathology department. Patients with concomitant bronchial asthma or allergic diseases were not excluded from the study in order to present the clinical picture of the disease as representatively as possible for the Western population. Patients with malignant diseases (e.g. cancer or inverted papilomma), fungal infection or other chronic inflammatory diseases were excluded from this study.

### ALI culture

Immediately after removal, the inflamed mucosa (*n* = 2) and nasal polyp tissue (*n* = 2) were put into 1x Dulbecco’s PBS (Capricorn, Germany) stored on ice and transported to the cell culture laboratory. There, excess connective tissue and coagulated blood were removed. The mucosa was cut into small pieces (approx. 2 mm³) and digested with Collagenase (0.375U/mL in PBS, NB4; Nordmark Pharma GmbH, Germany) at 37 °C for 1 h. If necessary, an erythrocyte lysis buffer solution (155mM NH4Cl, 10mM KHCO3, 0.1mM EDTA, pH 7.3) was applied after centrifugation of the suspension and decantation of the collagenase. The centrifuged cells were resuspended in PneumaCult™-Ex Plus medium (STEMCELL Technologies Inc., Canada), containing 10% (v/v) PneumaCultTM-ALI 10X Supplement, Penicillin G (10,000U/mL, Capricorn, Germany), Streptomycin (10,000 µg/mL, Capricorn, Germany), Amphotericin B (25 µg/mL), 4 µg/mL Heparin, 1µM Hydrocortisone (HC), and cultured for 3–7 days in T25 cell culture flasks (Starlab, Germany). Medium was changed every two days. After this pre-cultivation, the cells were detached with Accutase (Capricorn, Germany), seeded at a density of 10^5^ cells/cm² on 0.4 μm pore transwell inserts (9310412; SABEU GmbH & Co. KG, Germany) and incubated at 37 °C and 5% CO2. After 2–3 days, the cells reached confluence and were cultured at the ALI. The medium was switched to PneumaCult™-ALI medium (STEMCELL Technologies Inc., Canada) and changed every two days. After 14 days, the cells started to produce mucus, which was removed by washing the apical chamber with 1x PBS at each medium change. After 21 days, the cells showed morphological features of the respiratory epithelium, such as cilia, and were used for further experiments. The cultures derived from CRSwNP were termed SP-ALI and the ones from CRSsNP were named NS-ALI cultures.

### Nasal derived fibroblasts

For the establishment of primary fibroblast, biopsies from patients with CRSwNP (SPDF; *n* = 4)) and nasal mucosa from patients with CRSsNP (NSDF; *n* = 4)) were cut into small pieces and then digested with Collagenase and precessed as described above. Cells were resuspended in Dulbecco’s Modified Eagle Medium (DMEM) containing Penicillin G (10,000U/mL), Streptomycin (10,000 µg/mL), Amphotericin B (25 µg/mL), 2mM L-Glutamine and 10% Fetal calf serum (FCS) (Capricorn, Germany) and cultured in a T25 cell culture flask (Starlab, Germany) at 37 °C and 5% CO_2_. After two days of cultivation, the cells were washed with 1x PBS to remove non-adherent cells. The medium was changed every two days and the cells were grown to confluence. The purity of the fibroblasts obtained was determined by the characteristic spindle and bipolar or multipolar morphology and absence of epithelial colonies. For the subsequent experiments, the cells were used up to the 4th passage to ensure consistent and reliable results.

### Transepithelial electrical resistance (TEER) measurement

To assess epithelial integrity, each well of the respective ALI cultures were evaluated from day 2 to day 30 using TEER measurements, utilizing an Volt/Ohm Meter (EVOM2) equipped with the STX4 electrode (WPI, USA). To avoid contamination, the electrode was sterilized in 70% isopropanol for 15 min before each measurement. The medium in the basal chamber was completely removed and replaced with 2000 µl of TEER buffer at room temperature (HEPES 20mM, 0.9% NaCl, and 3mM CaCl₂), while 700 µl of TEER buffer at room temperature was added to the apical chamber. An blankinsert without cell culture served as the blank control. For TEER measurement, the electrode was placed in a free-standing position, with the longer electrode submerged in the lower chamber and the shorter electrode in the upper chamber. The TEER values were subsequently recorded. The TEER value was calculated using the following formula.The membrane area was 1.1 cm². Blank values were obtained immediately prior to TEER assessment and subtracted from the respective raw resistance values to account for background resistance of the membrane and TEER buffer. Data were analyzed *with GraphPad Prism (GraphPad Software, United States).*

$$\begin{aligned}&TEER\:(\Omega*cm^2)=(measured\:TEER\:value\:(\Omega)-blank\:value (\Omega))*membrane\:area\:(cm^2)\end{aligned}$$

### Migration and wound healing assay

Fibroblasts (SPDF *n* = 3; NSDF *n* = 3))were seeded in a 6-well with a density of 5 × 10^4^ cells each and allowed to grow to confluence. A 1000µL pipette tip (TipOne^®^, Starlab, Germany) was used to scratch a cross into the cells. Subsequently, the wells were rinsed with PBS (Capricorn, Germany) to remove detached cells. Cells were incubated for 48 h in DMEM with 10% FCS. After 6 h, 18 h, 24 h, and 48 h, the same 4 areas within three wells were imaged using a phase contrast microscope (Zeiss, Germany) and 10x objective. An ImageJ/Fiji^®^ plugin “Wound Healing Size Tool” (Suarez-Arnedo et al., 2020), was used to quantify the wound area. For migration analyses, the initial wound area boundaries of the scratches of 6 h were marked by a line for each image. Cells located between the boundaries were counted manually. The measurements from the four areas of each well were averaged to obtain a representative value. Data analysis was executed with GraphPad Prism (GraphPad Software, United States). For statistical analysis an unpaired t-test with Welch´s correction was used.

### Stimulation with inflammatory mediators

Stimulation of the respective ALI cultures require their weaning from HC, since HC would suppress the inflammatory reaction and could therefore influence the investigation of this reaction. For this purpose, the transwell inserts were washed twice with 1x PBS, transferred to a new 12-well plate and incubated for three days in ALI medium without HC. All stimulations of the cell lines were done in ALI medium without HC. Before each treatment the ALI cultures were washed with 1x PBS, to remove mucus.The stimulations with Poly(I: C) (HMW) (Invivogen, France) were performed in concentrations of 0 to 10 µg/mL for 24 h. For this, the stock solution containing 1 mg/mL of Poly(I: C) in 0.9% NaCl was heated to 65–70 °C for 10 min, cooled down for 5 min at room temperature and then applied directly to the basal chamber (culture medium) in the desired quantity, whereupon subsequently 50µL out of the basal chamber was applied to the apical chamber. Treatment with LTA and LPS was also carried out in the same method at concentrations of 10 ng/mL over the course of one day. The cells were then lysed with lysis buffer for qPCR analysis. Fibroblasts were seeded in a 6-well plate at a density of 5 × 10^4^ cells per well in DMEM with 10% FCS and allowed to grow until confluence. Subsequently, the cells were transferred to serum-free DMEM and starved for 24 h. To investigate the response of the respective nasal fibroblasts to the Th2 key mediators IL-4 and IL-13, the following conditions were applied: IL-4 alone, IL-13 alone, Dupilumab alone, IL-4 + Dupilumab, IL-13 + Dupilumab. For experiments using IL‑4 or IL‑13 alone, fibroblasts from *n* = 4 SPDF donors and *n* = 4 NSDF donors were analyzed. For experiments involving Dupilumab (Dupilumab alone, IL‑4 + Dupilumab, IL‑13 + Dupilumab), fibroblasts from *n* = 1 SPDF donor and *n* = 1 NSDF donor were used. In order to ensure optimal fibroblast response to IL-4 and IL-13, concentrations of 1ng/mL, 10ng/mL, and 50ng/mL were tested (Suppl. Figure 3). The optimum concentration for IL-4 was determined to be 1ng/mL and for IL-13 10ng/mL. Dupilumab was used in a concentration of 10 µg/mL. All experiments were conducted under serum-free conditions for 24 h.

### Growth curve

To determine differences in cell growth between the respective fibroblasts (SPDF *n* = 3; NSDF *n* = 3), 5 × 10^4^cells were seeded in three wells of a 6-well plate in DMEM + 10% FCS. Every second day, the cells were detached using Accutase, the cell count was determined under the microscope using trypan blue and a Neubauer cell chamber by double determination, and 2.5 × 10^4^cells were reseeded into the wells. The cell counts of the respective donors were analyzed and visualized using GraphPad Prism (GraphPad Software, USA). For statistical analysis, an unpaired t-test with Welch’s correction was used.

### Quantitative real-time polymerase chain reaction (qRT-PCR)

Total RNAs were extracted using the innuPREP RNA Mini Kit in compliance with the manufacturer’s guidelines (Innuscreen GmbH, Germany). The quality and quantity of isolated RNA were determined by a nanophotometer (Implen GmbH, Germany). For cDNA synthesis, 1 µg of RNA was processed with random hexamer primers using the Biozym cDNA kit (Biozym Scientific GmbH, Germany) according to the manufacturer’s instructions. If the RNA concentration was below 87ng/µL, the maximum volume of 11.5µL template was used. Real-time PCR was performed according to the manufacturer’s protocols of the Luna^®^ Universal qPCR Master Mix (New England Biolabs, USA). According to the user’s manual, the cDNA was used at a dilution of 1:10, and the primer concentration was set to 0.25µmol. All primers for targets used in this study are listed in Table 1. As endogenous control in each sample, glyceraldehyde-3-phosphate (*GAPDH*) was used. Each reaction was performed in triplicate with 40 cycles, 95 °C for 15s, and 60 °C for 30s using the MIC qPCR cycler (BioMolecular Systems, Australia). Relative expression levels were calculated using the 2^−ΔΔCT^ method, based on technical triplicates for each biological replicate. To ascertain the differences in the relative expression, fold-changes of the respective mean values were calculated. Graphics and statistical analysis were created with GraphPad Prism (GraphPad Software, United States). For statistical analysis an unpaired t-test with Welch´s correction or Mann-Whintey test was used.

**Table 1:** Overview of all primers, their targets, and nucleotide-sequence used for qRT-PCR. The primers were designed to have an amplicon size between 70 and 200 bp and an annealing temperature close to 60 °C

CCL11	AACCAGAGCCTGAGTGTTGC	IL33	CATGCCAACAACAAGGAACA
	AAACCCATGCCCTTTGGACT		AGGACAAAGAAGGCCTGGTC
CCL26	CCTGGGTGCGAAGCTATGAA	INF-γ	TGCAGGTCATTCAGATGTAGC
	CCTCTTTTGGTAGTGAATATCACAG		TCTTTTGGATGCTCTGGTCA
CLDN1	AGCTGTTGGGCTTCATTCTC	MMP-2	ATCCAGATTCCTCAGGCGG
	CTGGGCGGTCACGATGTT		TCCTGGCAATCCCTTTGTATGT
COL1A1	CCTGGGGCAAGACAGTGATT	MMP-9	TCCCTGGAGACCTGAGAACC
	AACGTCGAAGCCGAATTCCT		CCCGAGTGTAACCATAGCGG
FN1	CGCCGAATGTAGGTGAGGAA	POSTN	AAGAAGACACACCCGTGAGG
	GGCATTTGGATTGAGTCCCG		ACGACCTTCCCTTAATCGTCT
FOXJ1	CTGGGGCATAAGCGCAAACA	TLR2	AGATGCCTCCCTCTTACCCATGTT
	GTTGCCTTTGAGGGGTTCCA		AAGACTTTGGCCAGTGCTTGCT
GAPDH	CTGCACCACCAACTGCTTAG	TLR3	CATCCTCCACCACCAAGTGC
	GTCTTCTGGGTGGCAGTGAT		GCGGCTGGTAATCTTCTGAGT
Ki-67	AGTGCTGATGGTTTACAGGGG	TLR4	CACAGACTTGCGGGTTCTACATCA
	AGACTCCACGTCTCTTCCCT		TGGACTTCTAAACCAGCCAGACCT
IL-4	ACTGCTTCCCCCTCTGTTCT	TNF-α	CAAGGACAGCAGAGGACCAG
	CTGCTCTGTGAGGCTGTTCA		TCCTTTCCAGGGGAGAGAGG
IL-8	TCTCTTGGCAGCCTTCCTGATTTC	TSLP	GGCAAAACCTGGTGCTTGAG
	AGTTTTCCTTGGGGTCCAGACAGA		AGGACAAAGAAGGCCTGGTC
IL-13	CCACGGTCATTGCTCTCACT	Vimentin	GGACCAGCTAACCAACGACA
	CCGACACTCACCTTCTGGTT		AAGGTCAAGACGTGCCAGAG
IL25	CTGGAGGCTGGTCCCTTTTT		
	CTGCTCCAGACAGCACTTCA		

## Results

### Epithelial barrier maturation

**Fig. 1:**
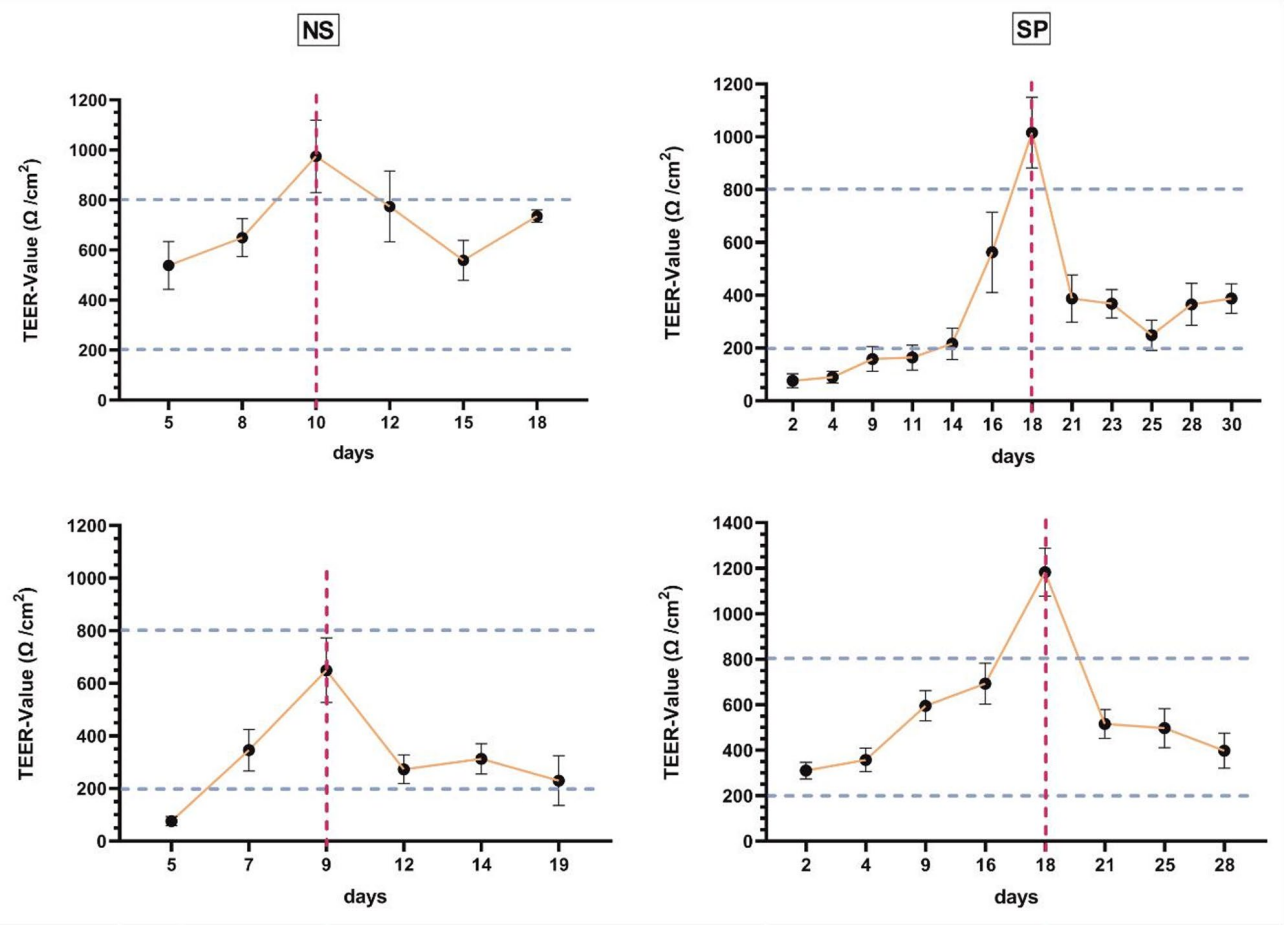
Evaluation of the in vitro establishment of the epithelial barrier by TEER measurements. Depicted are TEER measurements of NS (*n* = 2) and SP (*n* = 2) cultures. The TEER measurement of differentiation starts on day 2 after induction and includes a maximum (red dotted line) followed by a sustained constant resistance between 800 and 200 Ω/cm^2^ (blue dotted lines). Each point represents one measurement of all wells of the respective ALI culture at one timepoint ± SD NS cells peak at approximately 9–10 days and are fully mature at 18 days. SP cells peak after approximately 18 and also require approximately 10 days longer until reaching a plateaued resistance

The epithelial integrity of the respective ALI cultures during their differentiation was determined by several approaches. Both cell types formed a differentiated pseudostratified epithelium after 21 days consisting of ciliated, goblet and basal cells accompanied by the expression of distinct (suppl. Figure 1 A). A closer examination of the histology of the ALI cultures suggests that SP-derived ALI cultures exhibited stunted and irregular cilia formation (Suppl. Figure 1B). Comparing the development of epithelial barrier integrity in SP and NS-ALI cultures via TEER measurements, a clear difference in the time course of tight junction formation and maturation was observed. While the NS-ALIs reached their tight junction peak at day 9 (red dotted line, mean TEER = 1099,27 ((Ω/cm²)), the SP-ALIs showed a delayed differentiation of the tight junctions, peaking only at day 18 (mean TEER = 812,24 (Ω/cm²)). After 12 days, NS-ALIs achieved a sufficiently stable resistance of 200–800 (Ω/cm²) in the TEER measurement. In contrast, SP-ALIs required around 10 days longer (at day 18) to reached the consistent TEER plateau (Fig. 1) (Fig. 1). Analysis of the difference between tight junction peak and the plateau resulted in an average decrease of 647.52 (Ω/cm²) for the SP ALIs (day 18- day 21), whereas the NS ALIs only showed an average decrease of 288.66 (Ω/cm²) (day 10- day 12; day 9-day 12).All in all, basal epithelial stem cells derived from chronic inflamed nasal mucosa as well as NP have the potential to mature into functional pseudostratified epithelium in an ALI culture. However, it should be emphasized that SP-derived ALI cultures showed a significantly slower formation and delayed maturation of tight junctions and a less structured, rather abnormal distribution of cilia compared to NS-ALI cultures.

**Fig. 2:**
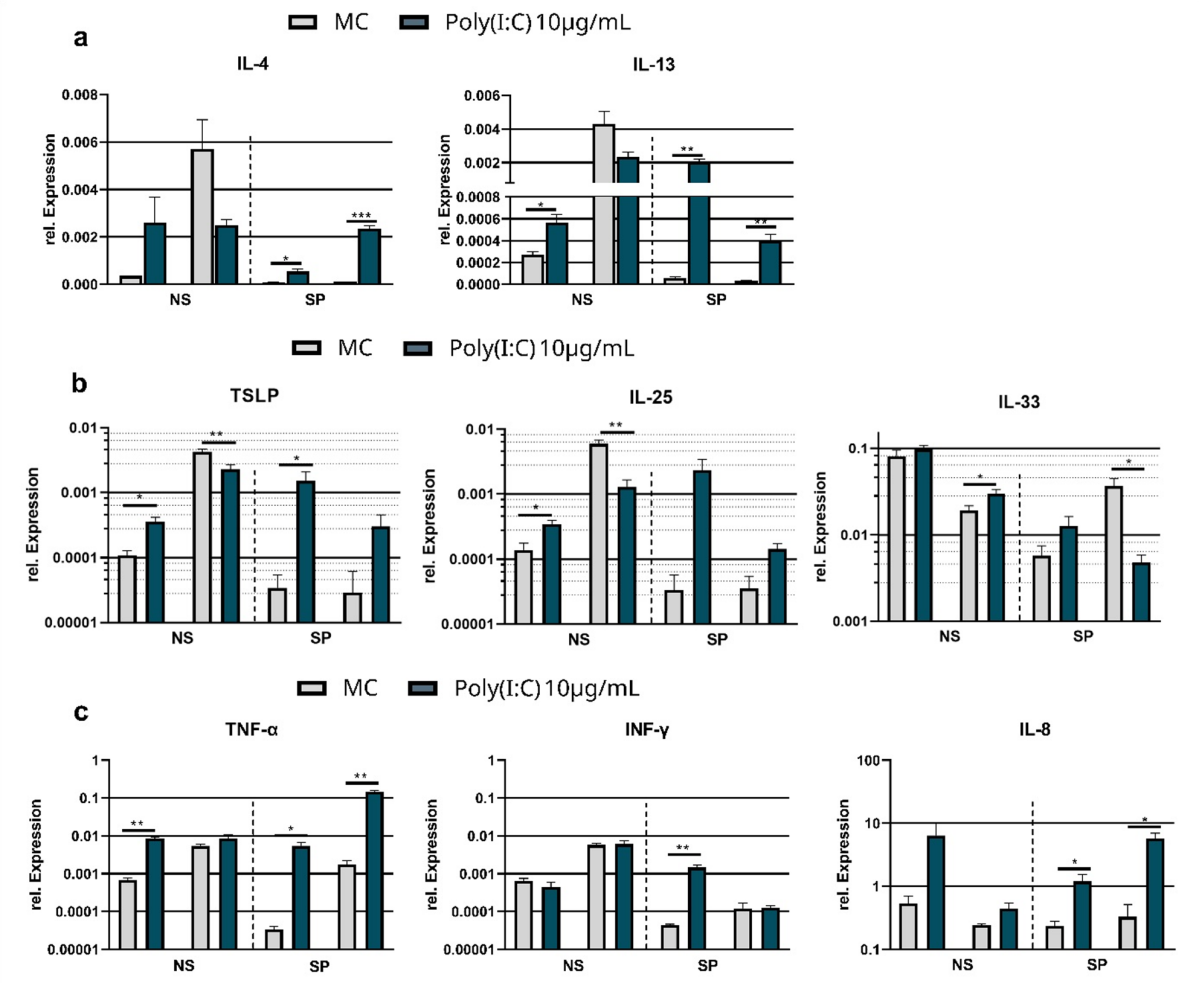
The expression of Th1/Th2 related mediators in ALI cultures derived from the respiratory epithelium of CRSsNP (*n* = 2) and CRSwNP (*n* = 2) after exposure with TLR3 agonist Poly(I: C), normalized to GAPDH. (**a**) The expression of IL4 and IL-13 was consistently induced after 1 day of TLR3 stimulation in SP cells with 10 µg/mL Poly(I: C). The relative increase in expression was also much higher in SP cells. (**b**) Treatment with Poly(I: C) led to a significant increase in gene expression of TSLP and IL-25 particularly in SP cells, where a consistent and reproducible increase in expression was observed. In contrast, IL-33 showed high variability in the results and failed to provide consistent, reproducible expression patterns. (**c**) Expression of Th1 related genes IL8, TNF-α and INF-γ. The expression of IL-8 is upregulated by the addition of Poly(I: C) to the medium in both NS and SP cells. TNF-α exhibited a significant and reproducible increase in expression exclusively in SP cells. In contrast, the expression of INF-γ showed no reproducible effect (Welch´s test, two Tailed, 95% confidence interval, * *p* < 0.05, ** *p* < 0.01, *** *p* < 0.001)

Investigations of Th1 and Th2 associated cytokines and chemokines in NS and SP derived ALI cultures upon stimulation with TLR3 agonist Poly(I: C) lead to a varying upregulation of Th2 associated mediators IL-4, IL-13, IL25, IL33 and TSLP. The highest expression of Th2-associated genes was observed in the SP-ALI cultures, with IL-13 demonstrating a clearly significant upregulation with a fold-change between 12.65 and 35.85 (*p* = 0.006, *p* = 0.002), followed by TSLP with an increased expression between fold-change of 14.3 and 44.1 (*p* = 0.229, *p* = 0.043). IL-25 was also strongly induced, with a change of 3.9- to 67.5-fold (*p* = 0.065, *p* = 0.069). IL-4 showed an significant increase in expression between 7.2 and 21.5 (*p* = 0.0118, *p* = 0.0009). In contrast, IL-33 expression did not displayed any trend in the SP-ALI cultures (Fig. 2A and B).

In contrast, only minor and non-reproducible changes were observed in the NS-ALI cultures. Fold changes for IL-4 were only between 0.4 and 7.3, for IL-13 between 0.6 and 2.1 (*p* = 0.0116). TSLP expression in NS-ALI cultures exhibited a moderate to small increase in expression with a fold-change of 0.5 to 3.3-fold (*p* = 0.005, *p* = 0.01), while IL-25 expression was only minimally induced with a fold-change of 0.2 to 2.5-fold (*p* = 0.0062, *p* = 0.043). Overall, the SP-ALI cultures showed a more pronounced and consistent upregulation of Th2-associated genes compared to the NS cultures.

To investigate how Poly(I: C) regulates Th1-related genes, the expression of TNF-α, IFN-γ, and IL-8 was analyzed (Fig. 2C). The most prominent effect was observed for TNF-α: in SP-ALIs, gene expression increased significantly and reproducibly by 81.9- to 155.5-fold (*p* = 0.0016, *p* = 0.016), whereas in NS-ALIs, the increase was more modest, ranging from 1.5- to 12.4-fold (*p* = 0.0031; *p* = 0,6967). For IFN-γ, a substantial 33.2-fold upregulation was observed in one sample of SP-ALIs(*p* = 0.007), while another sample showed no change (*p* = 0.916). In NS-ALIs, IFN-γ expression remained largely unaffected by Poly(I: C), with fold-changes between 0.6 and 1.1 (*p* = 0.137; *p* = 0.696). IL-8 expression was reproducibly induced in both cell types. While NS-ALIs, displayed an increased expression by 1.8- to 11.8-fold (*p* = 0.115; *p* = 0.134), SP-ALIs showed a stronger response, with increases ranging from 5.1 to 17.3-fold (*p* = 0.035, *p* = 0.015).

Importantly, the expression levels of other TLRs, namely TLR2 and TLR4, were at least one order of magnitude lower compared to TLR3. Moreover, stimulation of these receptors did not induce regulation of Th1 or Th2 cytokines in ALI cultures derived from either NS- or SP-ALIs, in contrast to the effects observed with TLR3 (Suppl. Figure 5 and Suppl. Figure 6).

### Altered migration capabilities of CRSwNP-derived fibroblasts

**Fig. 3:**
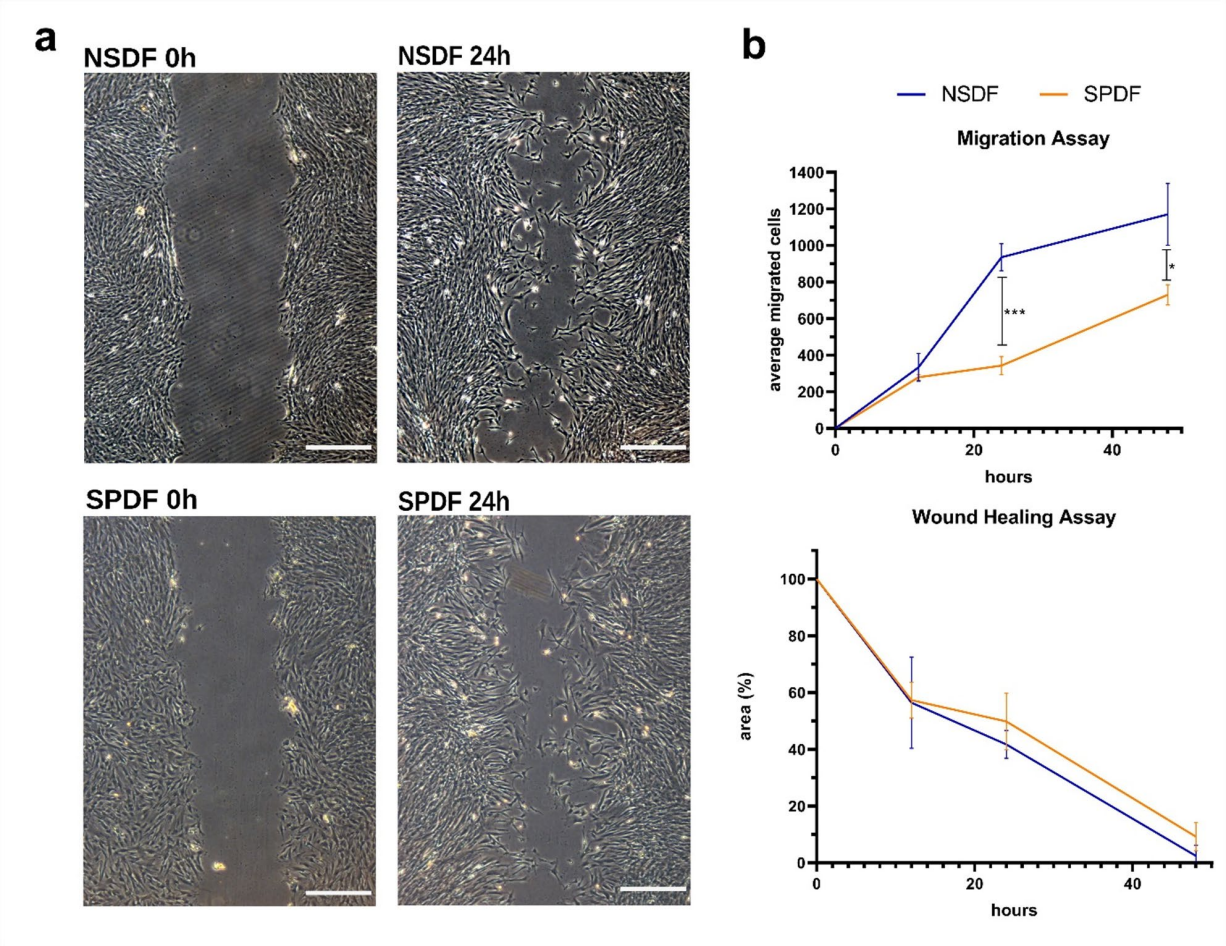
Investigations of the wound healing rate and migration characteristics of NSDF (*n* = 3) compared to SPDF (*n* = 3). (**a**) SPDF exhibited a rather limited ability to migrate into the gap compared to NSDF. (**b**) On average, almost 3 times as many NSDF as SPDF could migrate into the wound gap within 24 h, indicating a potentially increased migration capability (Welch´s test, two Tailed, 95% confidence interval, * *p* < 0.05, *** *p* < 0.001; scalebar=50 μm)

To detect differences in migratory behavior between the fibroblasts, a wound healing assay was performed. After 24 h, an average of 930 NSDFs had migrated into the wound gap, compared to only 340 cells for SPDFs, corresponding to a highly significant difference of 2.7-fold (*p* = 0.0006). Wound closure after 24 h was also higher, with NSDFs reaching 58.3% while in SPDFs only 50.2% of the wound was closed - a relative divergence of 16.1%. After 48 h, 1,170 NSDFs had migrated into the wound gap compared to 730 SPDFs, corresponding to a significant 1.6-fold higher migration rate in NSDFs (*p* = 0,0349). Consistently, wound closure was more advanced in NSDF cultures, with 97.6% of the wound area closed, whereas SPDF cultures achieved 91% closure. Overall, these findings indicate that NSDFs contribute more efficiently and rapidly to wound healing than SPDFs (Fig. 3).

### IL-4/IL-13 can promote pathogenic activation of CRSwNP fibroblasts

**Fig. 4:**
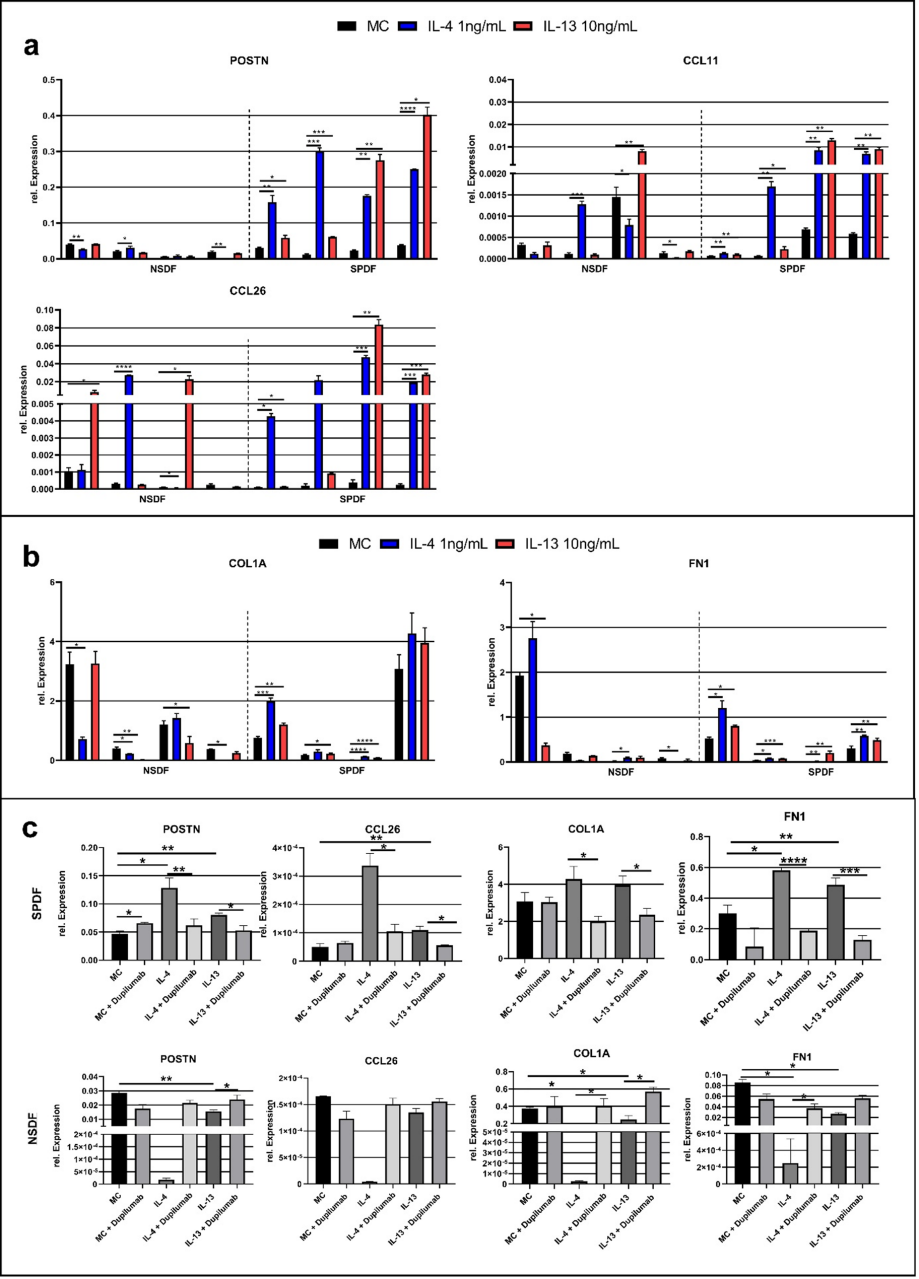
Comparison of the gene expression of IL-4/IL-13 treatment on NSDF (*n* = 4) and SPDF(*n* = 4) and the effects of Dupilumab (respective *n* = 1), normalized to GAPDH. (**a**) Treatment with IL-4 and IL-13 exhibited marked effects on the expression of POSTN, CCL11 and CCL26 in SPDFs and NSDFs. Overall, SPDF showed increased and more consistent expression of these genes, while only sporadic increases were observed in NSDF. Notably, IL-4 induced a marked increase in POSTN gene expression in SPDF. In contrast, CCL11 and CCL26 showed the tendency to be more inducible in SPDFs but no coherent significant change in their expression could be observed in either cell type in response to IL-4 or IL-13. (**b**) Comparison of gene expression of COL1A and FN1 in NSDFs and SPDFs. Treatments with IL-4 or IL-13 indicated a reproducible increase in COL1A and FN1 expression in SPDFs compared to NSDFs. Neither COL1A nor FN1 could be reproducibly increased by IL-4 or IL-13 in NSDFs. (**c**) Effect of Dupilumab on the gene expression of POSTN, CCL26, COL1A, and FN1 Fibroblasts were treated with Dupilumab alone, IL-4 alone, IL-4 + Dupilumab, IL-13 alone or IL-13 + Dupilumab. SPDF as well as NSDF display a significant restoration of the initial expression level. (Welch’s test, two Tailed, 95% confidence interval, * *p* < 0.05, ** *p* < 0.01, *** *p* < 0.001, **** *p* < 0.0001)

Stimulation of the fibroblastic cells with IL-4 and IL-13, common in the type 2 environment, showed that SPDF had a significantly stronger and reproducible response compared to NSDF (Fig. 4A). IL-4 stimulation led to a marked increase in CCL26 expression in SPDF ranging from 41- to 120.9-fold (mean fold-change: 85.4), while IL-13 showed a more variable induction between 1.3- and 213-fold (mean fold-change: 84.4). In NSDF, IL-4 showed inconsistent regulation between 0.02- and 89-fold (mean fold change: 22.6) and IL-13 also resulted in inconsistent expression ranging from 0.5- to 190-fold (mean fold change: 49.9). For the chemokine CCL11 an significant upregulation in SPDF by IL-4 was distinctively and was in the range of 2 to 24.9-fold (mean fold-change: 12.7), whereas IL-13 induced a comparable induction of 1.5 to 18.7-fold (mean fold-change: 10.5). In NSDF, regulation of CCL11 by IL-4 was limited to 0.2 to 11.3-fold (mean fold-change: 3.1), also IL-13 induced only a change between 0.96 and 5.6-fold (mean fold-change 2.2). IL-4 induced increased POSTN expression in SPDF in the range of 5.2- to 26.3-fold (mean fold-change: 11.3), whereas in NSDF no consistent and significant increase of 0.001- to 1.5-fold was observed (mean fold-change: 0.9). Similarly, stimulation with IL-13 in SPDF resulted in an increased expression between 1.9- and 11.5-fold (mean fold-change: 7.4), whereas in NSDF a constant expression in the range of 0.8- to 1-fold was measured (mean fold-change: 0.9). Overall, upregulation of POSTN by IL-4 or IL-13 revealed a significant difference in SPDF compared to NSDF (Mann-Whitney *p* = 0.028). Considering the expression of extracellular matrix transcripts in SPDFs, IL-13 resulted in a drastic increase in gene expression of FN1 between 1.5-fold and 33.6-fold (mean fold-change: 9.7), while IL-4 induced a significant, albeit less variable, increase in SPDF ranging from 1.93- to 2.8-fold (mean fold-change: 2.27). In NSDFs, a non-consistent change in expression of FN1 between 0.0009-fold and 3.9-fold was observed through treatment with IL-4 (mean fold-change: 1.37), while IL-13 resulted in heterogenic regulation of 0.5- to 190-fold (mean fold-change:49.9). In SPDF, IL-4 led to an increase in COL1A expression in the range of 1.4 to 9.7-fold (mean fold-change: 3.6), while IL-13 caused a comparable upregulation between 1.4 and 9.7-fold (mean fold-change: 2.49). In NSDF, however, stimulation with IL-4 led to a downregulation with values of 0.00007- to 1.2-fold (mean fold-change: 0.5); IL-13 also exhibited a downregulation of between 0.07- and 1-fold (mean fold-change: 0.55). To investigate the therapeutic effect of Dupilumab treatment on the expression of POSTN, CCL26, COL1A, and FN1 in the respective fibroblasts was then systematically analyzed (Fig. 4C). Even if the SPDF treated with IL-4 showed a comparable low 2.7-fold increase in POSTN expression, the treatment with Dupilumab led to a significant reduction to 0.5-fold and thus to the original expression level. In IL-13-treated SPDF, a 1.7-fold increase in POSTN expression was observed, with the combination with Dupilumab reducing POSTN expression by 1.5-fold to the level of the untreated control group. CCL26 expression was significantly elevated 6.6-fold in IL-4-treated SPDF and was decreased 3.2-fold to near control levels in Dupilumab treated SPDF. A 2-fold decrease in CCL26 expression was reported in Dupilumab-treated SPDF, which was very similar to the control group. COL1A expression was increased 1.3-fold in IL-4-treated SPDF compared to the control group, while treatment with Dupilumab resulted in a 2.1-fold decrease in expression. IL-13-treated SPDF exhibited a 1.2-fold increase in expression, which decreased 1.7-fold following Dupilumab treatment. Investigations of FN1 expression revealed that SPDF treated with IL-4 exhibited a 1.4-fold increase, while treatment with Dupilumab exhibited decreased expression by 3-fold.

## Discussion

### Nasal polyp derived basal stem cell exhibited late differentiation

Defects in the physical barrier can arise from immunological factors such as allergic, microbial, or viral triggers, as well as from conditions like hypoxia or zinc deficiency. Notably, a compromised physical barrier is a key hallmark of CRS pathogenesis [33]. Comparing only the final TEER values and cellular composition of matured ALI cultures (Fig. 1 and Suppl. Figure 1), both parameters converged irrespective of the source, typically reaching ~ 200 Ω/cm²—consistent with values reported for differentiated respiratory epithelium [34]. Besides that, TEER development diverged markedly between SP-ALI and NS-ALI cultures, with NS-ALI showing a rise by day 10, while SP-ALI lagged behind until day 18—contrasting with reports of peak TEER as early as day 2 in epithelial cells from healthy donors or CRSsNP patients [35]. From studies of the establishment of human bronchial ALI cultures it is known, that a tightly packed cell structure and the formation of tight junction proteins occurs in the first week after induction, which causes the initial increase in the TEER value [36, 37]. It seems, that the SP-cultures are impaired in their normal progression at this stage, in accordance this was observed in vivo where they were not able to express sufficient amounts of tight junction proteins in a reasonable time [38] and more importantly in ALI-cultures in vitro [39]. Transcriptomic and mRNA–miRNA network analyses of CRSwNP-ALI cultures revealed broad downregulation of genes linked to cell cycle regulation, proliferation, and ciliogenesis. Notably, key ciliogenesis-related microRNAs, including the miR-34 and miR-449 families, were significantly reduced, indicating impaired epithelial renewal and ciliary differentiation in CRSwNP [40]. In accordance with that, Yu et al. demonstrated that human nasal epithelial stem/progenitor cells (hNESPCs) from patients with NP have a significantly lower growth and proliferation rate compared to healthy patients [41]. The final TEER value of the mature state was already reached around day 12 for NS-ALI, but not until the day 21 or even day 30 for SP-ALI. (Fig. 1, Suppl. Figure 2). It is known, that from the second week onwards, a pseudostratified epithelium develops under ALI conditions and the differentiation of specific cell types begins. During this phase, the TEER value decreases as the cells develop their columnar morphology. In the third week, cellular differentiation into goblet and ciliated cells terminates, leading to a stable TEER.cm^2^ [42, 43]. An indication of this disturbed differentiation potential could be the architectural changes occurring in the epithelium of NP tissue, such as hyperplasia or metaplasia of the goblet and basal cells [44, 45]. The altered proliferative behavior may result in a prolonged time span required for the cells to become sufficiently tightly packed to reach the tight junction peak. Furthermore, we hypothesize that the pronounced decline from the tight junction peak to the plateau may be associated with an impaired differentiation potential of the SP-derived cells (Fig. 1). This cellular behavior might be transmitted into the in vitro cultivation thereby hindering the fast establishment of a new epithelial barrier. This indicates a limited extent of epithelial restitution in NP and suggests possible abnormal intrinsic differences in the hNESPCs of affected patients, which could be possible factors for the delayed differentiation of the ALI culture [41]. Studies investigating biopsies from patients with CRSwNP have demonstrated a reduced expression of tight junction proteins [46], suggesting that this tendency toward lower tight junction protein expression may delay tight junction maturation and consequently compromise epithelial integrity and functionality. Callejas and colleagues established an ALI model of NP and healthy nasal mucosa and performed comparative analyses between them. As observed in Suppl. Figure 1 their reconstituted epithelium showed morphological and functional characteristics very similar to those of the natural healthy nasal epithelium. Neither differences in cell composition nor changes in the tight junctions, the basal cell layer or hyperplasia of goblet cells could be detected during maturation. Unfortunately, no measurement of TEER values was provided in this study [47]. In contrast, another study comparing ALI cultures of healthy donors and NP revealed that SP-ALI-cultures retained features of their in vivo phenotype, including abnormal ciliary architecture and impaired differentiation, as shown by irregular and densely packed cilia in both native tissue and NP-derived cultures [48]. Interestingly an additional study with a similar cohort found an increased permeability of the epithelium in late stage of differentiation [49]; notably, an abnormal ciliary architecture was likewise observed in the histological analysis of the SP-ALI cultures (Suppl. Figure 1b). It is uncertain, if this contradicts the similar late stage TEER value reported here, because the comparison only took place between healthy donors and CRSwNP. A similar cohort design was used to study ALI cultures from asthma patients, which presented additional abnormalities compared to healthy subjects. These included patchy disruption of tight junctions, reduced TEER and increased permeability of the epithelium, indicating reduced epithelial integrity [50].

These results suggest that structural abnormalities of cilia are a characteristic feature of NP both in vivo and in vitro. To the best of our knowledge a prolonged differentiation duration of SP-ALI cultures has not been explicitly demonstrated to date. However, our data suggest that NP-derived hNESPCs may have intrinsic regulatory characteristics that could be related to the differentiated epithelium, which show signs reminiscent of the abnormal phenotype of in vivo polyps. This could be a basis for the persistence and abnormal remodeling in NP and could potentially play an important role in the development and maintenance of this pathological condition. Nonetheless, these observations are preliminary and require further investigation with higher donor number.

### Nasal epidermal cells react differently to Poly(I: C)

Immunological memory has long been attributed only to specialized immune cells such as T and B lymphocytes. Today it is known that prolonged exposure of sentinel cells e.g. macrophages to PAMP can also induce this kind of memory through chromatin modifications, which store information about previous infections and enable a faster reaction [51]. However, more recent findings demonstrated that non-immunological sentinel cells such as epithelial cells [52] can also develop such a “trained memory” through epigenetic rewiring. Persistent influence of damaging and/or inflammatory factors recognizable by pattern recognition receptors can cause these cells to maintain their pro-inflammatory status even after the acute inflammatory phase has subsided [53]. Therefore, TLR3 agonist Poly(I: C) was used to test the NS and SP-ALI cultures for their ability promote type 2 inflammation upon an viral infection. On average, SP-ALI cultures responded with an increased and reproducible expression of IL-4, IL-13, TSLP, IL33 and IL8 on Poly(I: C), which was not the case for the NS-ALI cultures (Fig. 2). TSLP, IL-25, and IL-33 are classified as alarmins, the main cytokines released by the respiratory epithelium in response to tissue damage or pathogen exposure able to trigger Th2-immune response [54, 55].

The induction of the two core cytokines of the Th2-mediated inflammation IL-4 and IL-13 upon stimulation of TLR3 was investigated in normal nasal epidermal stem cells [56]. This study revealed, that treatment with Poly(I: C) induced a significant increase in the secretion of IL-4 in primary nasal epithelial cells, but unfortunately no secretion of IL-13 could be detected. Anyway, we suggest, that NS-ALI cultures mostly derived from a Th1-mediating environment may have lost this property or the differentiation into respiratory mucosa has veiled this capability. In accordance with our study, Golebski et al. were able to show that, that Poly(I: C) is the only TLR agonist that can enhance TSLP expression in epithelia cells of NP.In contrast to our study, only a slight increase in expression of IL-33 was observed, but IL-25 could not be detected. Unfortunately, they only compared the polyp derived cells to healthy epithelial cells [57]. Stimulation with a TLR3 agonist significantly increased the expression of TSLP and IL-25, but not IL-33, in CRSwNP epithelial cells, whereas cells without NP showed little change. In addition, our data showed a lower baseline expression of TSLP in SP-ALI cultures, which may be due to epigenetic mechanisms, as increased DNA methylation at the TSLP locus was detected in CRSwNP patients, which is associated with disease development [58].

The upregulation of IL8 we could identify in our data, was also verified by previous studies, which demonstrated that TLR3 agonists led to an increased expression of IL-8 in human nasal polyp epithelial cells [59, 60].

In this context, viral infections are believed to create an inflammatory memory that sensitizes epithelial cells for a faster and more efficient response to subsequent infections. We hypothesized to result this in an increased expression of TLR receptors, a phenomenon we also observed in SP cultures (Suppl. Figure 6). Our findings align with this premise, as neither LPS nor LTA induced a reproducible increase in the expression of TSLP, IL-25, or IL-33 (Suppl. Figure 5 A) nor an increased expression of TLR2 and TLR4 was detected (Suppl. Figure 6) [57]. Similarly, Wang et al. proposed that Poly(I: C) exerts a stronger pro-inflammatory effect on the nasal epithelium compared to LPS or LTA, which failed to enhance TLR2 or TLR4 expression [59]. The respiratory nasal epithelium, which is constantly exposed to commensal bacteria, may maintain a controlled inflammatory state. This finely balanced system has evolved to respond to specific pathogens with a heightened inflammatory response, while remaining tolerant to non-pathogenic commensals, ensuring a balance between defense and tolerance [59, 61]. The relevance of virus infections is also highlighted by the fact, that clinical studies have revealed that viral infections are the most common triggers for the onset for exacerbations of CRS and can lead to inflammation of the sinus mucosa, disrupting the physical protective barrier of the sinus epithelium [62, 63]. Taken together, these observations could indicate a tendency toward oversensitive proinflammatory signaling in epithelial cells from nasal polyps. Future studies with larger sample sizes could help refine the results and uncover possible underlying mechanisms.

### Nasal polyp derived fibroblasts

Fibroblasts are essential cells in connective tissue, contributing to ECM synthesis, cytokine and growth factor secretion, and wound healing regulation [64, 65]. Under normal conditions, fibroblasts are recruited to inflammatory sites like NP through chemoattractants, where they proliferate [66]. Our focus was on identifying intrinsic differences regarding fibroblasts main characteristics between CRSsNP and CRSwNP. Using wound healing assays, we found that SPDFs migrated significantly slower than NSDFs, with wounds still open after 48 h (Fig. 3). While previous studies examined fibroblast migration under inflammatory stimuli, a direct comparison between CRSwNP and CRSsNP is missing [67–69]. It is known that MMPs play a critical role in cell migration and wound healing, and that dysregulation can lead to impaired wound healing and tissue remodeling [70–72]. Further investigations revealed that there was no significant difference in proliferation (Suppl. Figure 4 A) or myofibroblasts differentiation status and ECM production (Suppl. Figure 4 B) in the primary fibroblasts. However, it was noticeable that NSDFs exhibited only significantly higher expression MMP2 on average, but not in MMP9 (Suppl. Figure 4 C). This may be an explanation for the observed differences in migration since García-De-Alba et al. demonstrated that pharmacological inhibition of MMP2 and MMP9 in human fibrocytes led to a significant reduction in cell migration [73]. This effect was also observed in primary fibroblasts in other clinical context [74]. This observation suggests that the higher MMP expression in NSDFs may support better migration, and further functional studies are therefore needed to understand the underlying mechanism in more detail. Moreover this reduced migration capacity could possible result from long-term imprinting by inflammatory cues and persist in vitro, aligning with findings of delayed wound healing from primary nasal epithelial cells from CRSwNP patients, possibly due to intrinsic, genetically or epigenetically driven repair defects [75]. Furthermore, studies on keloid fibroblasts compared to healthy fibroblasts have revealed an altered program of DNA methylation and histone acetylations, which may influence the wound healing capabilities of fibroblasts [76]. From a clinical perspective, impaired wound healing in CRS patients may be highly relevant, as epithelial barrier dysfunction is associated with increased susceptibility to mucosal injury. Importantly, intranasal corticosteroids, which represent a cornerstone in CRS therapy, have been reported to induce micro wounds and epistaxis [77].

Fibroblasts, like epithelial cells, act as sentinel cells that recognize pathogens, initiate immune responses, and exhibit immunological memory upon repeated stimulation [78]. We analyzed fibroblasts from patients with and without NP, focusing on POSTN, eotaxins (CCL11, CCL26), and ECM proteins (COL1A, FN1) in response to IL-4 and IL-13. Our results showed that SPDFs had a significant, reproducible increase in target gene expression compared to NSDFs after stimulation.

Our data revealed that unstimulated SPDFs express similar levels of POSTN as NSDFs, but respond strongly to IL-4 or IL-13 stimulation (Fig. 4). Previous studies have reported significantly elevated POSTN levels in tissue and serum of NP patients, suggesting a potential role in CRSwNP pathogenesis [79–82]. It is known that POSTN is produced by epithelial cells, endothelial cells as well as fibroblasts in response to IL-4 and IL-13 [83]. We think, that the presence of IL-4 and IL-13 in NP might act on the resident fibroblast, resulting in the observed correlation between POSTN expression and polyps appearance. But this mechanism does not work in CRSsNP even if IL-4 or IL-13 would be present. Takayama and colleagues also demonstrated that both IL-4 and IL-13 can cause the expression and secretion of POSTN by fibroblasts from asthma patients and contribute to subepithelial fibrosis [22]. Our data suggests, that the same mechanism of IL-4 or IL-13 stimulated fibroblast promoting the fibrosis might also be present in NP.

CCL11 and CCL26 are eotaxins with potent chemoattractant properties for eosinophils that play a central role in the immune response and in particular in the promotion of Th2-mediated inflammatory reactions [84, 85]. The results demonstrated that both CCL11 and CCL26 are highly upregulated in SPDFs upon treatment with IL-4 or IL-13 in all analyzed cultures. In contrast, the increase in NSDF was smaller and not consistent trough out the investigated cultures (Fig. 4A). This finding is in accordance with Steinke and colleagues, who were able to show that both the expression and secretion of CCL11 could be increased by IL-4 in nasal polyp fibroblasts [86]. Similar results were already observed from Imoto et al. for CCL26 in nasal polyp fibroblasts [87]. Additionally, Yamada et al. demonstrated that IL-13-treated nasal polyp fibroblasts secrete CCL26, whereas CCL11 secretion was not detected [88].

Airway remodeling in CRSwNP is characterized by significant deposition of collagen (types 1, 3 and 5) and FN1 in the subepithelial extracellular matrix. These changes lead to tissue hardening and thickening of the basement membrane, which contributes to the deterioration of nasal breathing and mucociliary clearance [18, 19]. In our studies, we were able to reveal that SPDFs show a pronounced upregulation of COL1A expression under the influence of IL-4 and IL-13, whereas NSDFs demonstrate a downregulation of this collagen expression in comparison. Consistent with the findings for SPDF, transgenic mice showing overexpression of IL-4 in the skin exhibited a significant increase in collagen types 1, 3 and 5 compared to wild type mice [89]. Similar to our results, Lenardi and colleagues found that conjunctival fibroblasts isolated from patients with vernal keratoconjunctivitis, a type 2 inflammation driven disease, manifested increased collagen production (especially type 1) under the influence of IL-4 and IL-13 [90]. Likewise, fibroblasts from keloids, also driven by type 2 inflammation, revealed that an increase in collagen expression could be achieved by IL-4 and IL-13, but unlike to SPDF IL-13 clearly showed a stronger effect. More importantly, no significant difference in the expression of COL1A was seen compared to healthy fibroblasts in this study [91]. FN1 demonstrates no significant increase in expression in NSDFs by either IL-4 or IL-13, whereas in fibroblasts from NP IL-4 and IL-13 caused a slight upregulation of FN1. Postlethwaite and colleagues found that treatment of normal infant foreskin fibroblasts with IL-4 or IL-13 could increase the expression of FN1 [92]. Similarly, studies on healthy lung fibroblasts showed that both IL-4 and IL-13 positively regulate FN1 expression [93]. Our results point to the direction, that for nasal fibroblasts the FN1 expression follows the pattern of the healthy fibroblasts, but with an increased output particularly for SPDF. But in contrast to this, some studies on fibroblasts from NP revealed that IL-4 significantly reduces FN1 production, whereas IL-13 has no effect on the expression of this protein [86].

The molecular signaling events leading to the of the observed stimulation starts with IL-4 and IL-13 exerting their effect by binding to the membrane receptors IL-4R and IL-13Rα1. The fact that fibroblasts are capable of expressing this type of receptor is known and can therefore contribute substantially to the clinical picture of polyps [86, 94]. Studies demonstrated that IL4R is upregulated in NP, but not IL13Rα1, compared to healthy nasal mucosa [95]. Similarly, Bellini et al. found increased IL4R expression in fibrocytic cells of asthma patients. In contrast, our data indicate that IL4R expression is not upregulated in fibroblasts from patients with CRSwNP compared to those with CRSsNP (Suppl. Figure 7). Notably, fibroblasts derived from NP exhibit a markedly enhanced responsiveness to IL-4 and IL-13, despite the absence of a concomitant increase in IL4R expression. This observation suggests that the regulation of pro-fibrotic and pro-inflammatory gene expression in these cells is possibly governed by intrinsic mechanisms rather than by receptor abundance alone. Inhibition of IL-4R by Dupilumab may have reduced the expression of POSTN, CCL26, COL1A and FN1 in SPDFs, but not in NSDFs or CCL11 (Fig. 4C), indicating a possible specific blockade of the IL-4R signaling cascade and Th2 inflammatory response [30]. In dermal fibroblasts, IL-13 induced collagen production via STAT6 and PI3K, which was reduced by their inhibition [96] and STAT6 knockout mice showed decreased COL1 and FN1 expression [97]. These findings suggest that IL-4/IL-13 signaling via IL-4R and IL-13Rα1 plays a key role in regulating pro-inflammatory and profibrotic responses. Notably, the upregulation of IL4R in NP and asthma-associated fibrocytes indicates enhanced activation under pathological conditions. Overall, the data provide initial indications of the feasibility of IL-4R inhibition, e.g. by Dupilumab, for modulating selected fibrotic and inflammatory markers. However, due to the small sample size, these results allow only minimal conclusions to be drawn about causal mechanisms or therapeutic efficacy.

## Conclusion

The present data provide important evidence of functional differences between cells from patients with CRSwNP and those from patients with CRSsNP, particularly regarding differentiation, barrier function, inflammatory response and repair capacity. In vitro, hNESPCs from NP revealed a delayed differentiation under ALI conditions as well as a reduced and delayed formation of tight junctions, which could indicate a possible limited restoration of epithelial barrier function. In addition, CRSwNP derived epithelial cells responded more strongly to viral stimuli with an increased release of pro-inflammatory mediators, which could indicate an altered response that is possibly characterized by long-term environmental influences or disease processes. Fibroblasts from NP also exhibited increased reactivity to IL-4 and IL-13, resulting in increased expression of pro-fibrotic and inflammation-related genes. This could indicate that the inflammatory environment in the tissue has influenced cellular behavior, potentially contributing to the altered cellular function contributing to chronicity and tissue remodeling in CRSwNP.

Despite these promising insights, it should be emphasized that the results are based on a limited sample size and should therefore be considered preliminary. Further studies with larger patient cohorts, as well healthy controls are necessary to make substantiated statements about the cellular and molecular mechanisms of CRSwNP. Moreover epigenetic analysis should lay in the focus of future studies. These should also aim to characterize the underlying regulatory mechanisms of the observed phenotypes more comprehensively.

## Supplementary Information

Below is the link to the electronic supplementary material.

Supplementary Material 1: Supplementary Fig. 1 Analysis of transcriptional changes in the developing airway epithelium and its morphology (respective *n* = 1). (a) After 21 days of differentiation, a marked upregulation of the transcription factor FoxJ1, responsible for ciliogenesis, and of the tight junction protein claudin1 (CLDN1) can be observed in both NS and SP cells compared to day zero. (b) Microscopic characterization of ALI cultures using HE staining. Cross-sections of respiratory epithelium differentiated on ALI membranes for 21 days reveal a well-organized, pseudostratified structure with ciliated cells in NS-derived cultures, whereas SP-derived ALI cultures display stunted and irregular cilia formation (Welch´s test, two Tailed, 95% confidence interval, ** *p* < 0.01, *** *p* < 0.001, ****P < 0.0001,)

Supplementary Material 2: Supplementary Fig. 2 Evaluation of the physical epithelial barrier by TEER measurements. Depicted are the TEER measurements of SPALI-culture (*n* = 1) showing particularly slow differentiation behavior. The TEER measurement of differentiation started on day 21 after induction and the measurement was continued until day 48. Each point represents one measurement of all wells of the respective ALI culture at one timepoint. The ALI culture reveals a decrease starting at day 26 and settles on a constant resistance of 800 − 200 Ω/cm2 from day 30–48 onwards

Supplementary Material 3: Supplementary Fig. 3 Stimulation of SPDF (*n* = 3) with various concentrations of IL-4 and IL-13 (1ng/mL, 10ng/mL and 50ng/mL) to determine the optimal stimulation concentration. Fibroblasts were allowed to grow to confluence, weaned for 24 h under serum-free conditions, and then treated for 24 h. The expression changes of POSTN, CCL11 and CCL26 normalized to GAPDH were examined. For further experiments, a concentration of 1ng/mL was used for IL-4 and 10ng/mL for IL-13 (Welch´s test, two Tailed, 95% confidence interval, ** *p* < 0.01, *** *p* < 0.001, ****p < 0.0001 compared to MC)

Supplementary Material 4: Supplementary Fig. 4 Evaluation of growth, differentiation and migration-associated genes in NSDF (*n* = 3) and SPDF (*n* = 3). (A) Growth experiments of NSDF and SPDF) on average demonstrated no statistically significant differences in cell division (*p* = 0.513) or proliferation regarding Ki-67 expression (*p* = 0.63). (B) Investigations regarding differentiation status revealed no significant differences in the expression of vimentin (*p* = 0.258) and COL1A (*p* = 0.29). (C) Analysis MMPs expression revealed that NSDFs have significantly higher baseline expression on average of MMP2 compared to SPDFs (*p* = 0.008). However, MMP9 did not exhibited statistically higher expression in NSDF (*p* = 0.076). All expressions were normalized to GAPDH (Welch´s test, two Tailed, 95% confidence interval, ** *p* < 0.01)

Supplementary Material 5: Supplementary Fig. 5 Expression of key Th1 and Th2 mediators in ALI cultures derived from the respiratory epithelium of CRSsNP (*n* = 2) and CRSwNP (*n* = 2), normalized to GAPDH. (a) Neither LPS (TLR4 agonist) nor LTA (TLR2-agonist) were able to induce a consistent upregulation of the TSLP, IL-25 and IL-33 genes. In contrast a partial downregulation of these genes was observed in both cell types, NSDF and SPDF. (b) Treatments with 10ng/mL LPS resulted in no reproducible upregulation of either TNF-alpha and INF-gamma or IL-8 in CRSsNP and CRSwNP derived ALI cultures. (Welch’s test, two Tailed, 95% confidence interval, * *p* < 0.05, ** *p* < 0.01)

Supplementary Material 6: Supplementary Fig. 6 The expression of TLRs from NS (*n* = 2) and SP (*n* = 2) derived ALI cultures of nasal epithelial cells, normalized to GAPDH. The cells were treated with 10ng/mL LTA (TLR2 agonist), Poly(I: C) (TLR3 agonist) or LPS (TLR4 agonist) for 24 h and the expression of the respective receptors was analyzed. In NS- and SP-ALI, neither LTA nor LPS are able to upregulate their respective TLR, whereas Poly(I: C) is capable of upregulating TLR3 in SP-ALI. (Welch’s test, two Tailed, 95% confidence interval, * *p* < 0.05, ** *p* < 0.01)

Supplementary Material 7: Supplementary Fig. 7 Investigation of IL4R expression of NSDF (*n* = 4) and SPDF (*n* = 4) by treatment with IL-4 and IL-13. Overall, SPDF exhibited increased and more consistent expression of this receptor subunit, while no increases was observed in NSDF (Welch’s test, two Tailed, 95% confidence interval, * *p* < 0.05, ** *p* < 0.01)

## Data Availability

All data are provided in this study, and raw data can be requested to the corresponding author.
