# Supplementary figures and images for: CRSwNP-derived cells retain native disease-relevant characteristics in vitro
Source: J Inflamm (Lond). 2026 Mar 19;23:10. doi: 10.1186/s12950-026-00497-7 (PMC13063898; doi:10.1186/s12950-026-00497-7)

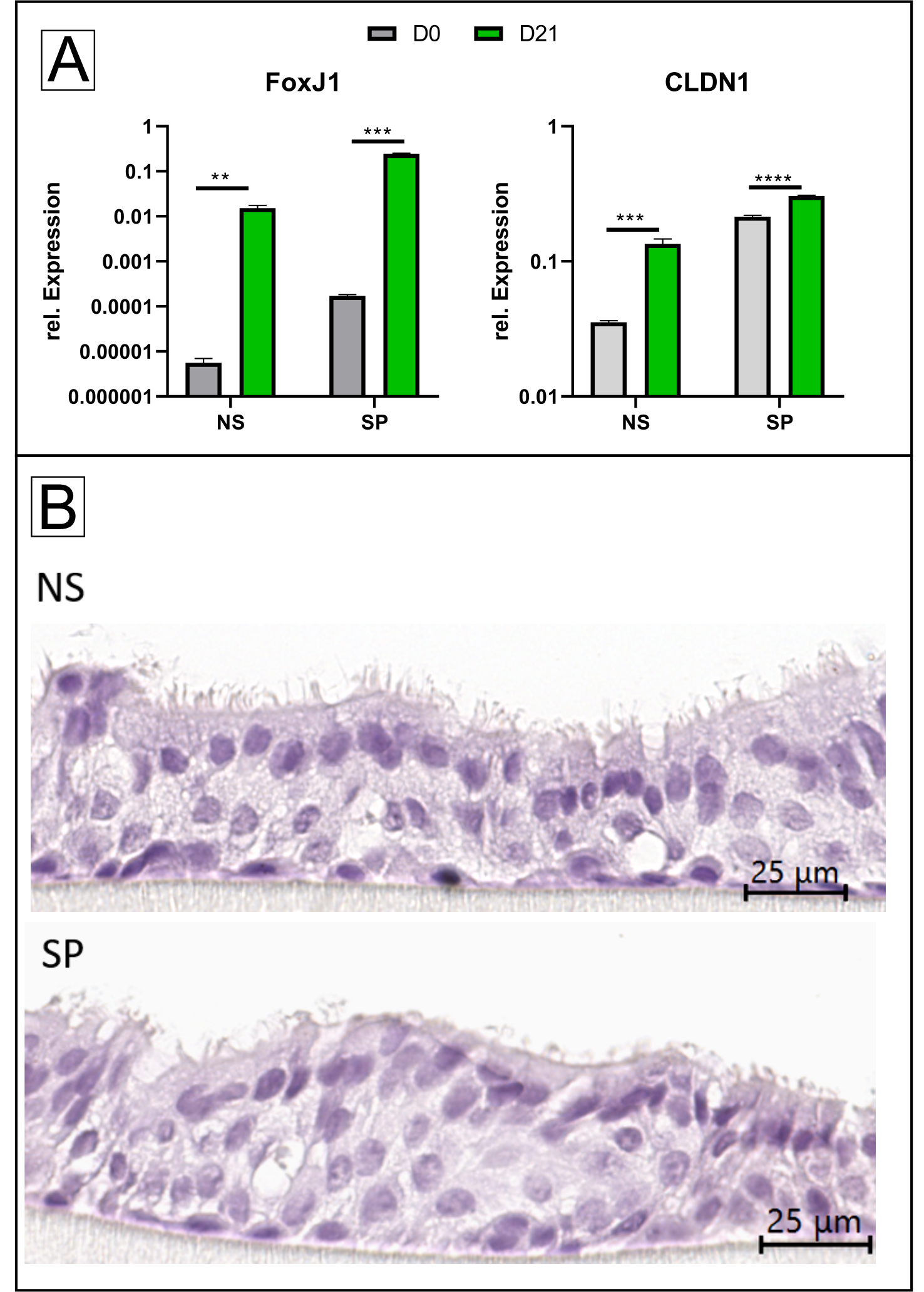

Supplement: Supplementary file 1 — Supplementary Material 1: Supplementary Fig. 1 Analysis of transcriptional changes in the developing airway epithelium and its morphology (respective n = 1). (a) After 21 days of differentiation, a marked upregulation of the transcription factor FoxJ1, responsible for ciliogenesis, and of the tight junction protein claudin1 (CLDN1) can be observed in both NS and SP cells compared to day zero. (b) Microscopic characterization of ALI cultures using HE staining. Cross-sections of respiratory epithelium differentiated on ALI membranes for 21 days reveal a well-organized, pseudostratified structure with ciliated cells in NS-derived cultures, whereas SP-derived ALI cultures display stunted and irregular cilia formation (Welch´s test, two Tailed, 95% confidence interval, ** p < 0.01, *** p < 0.001, ****P < 0.0001,) [file 12950_2026_497_MOESM1_ESM.tif]

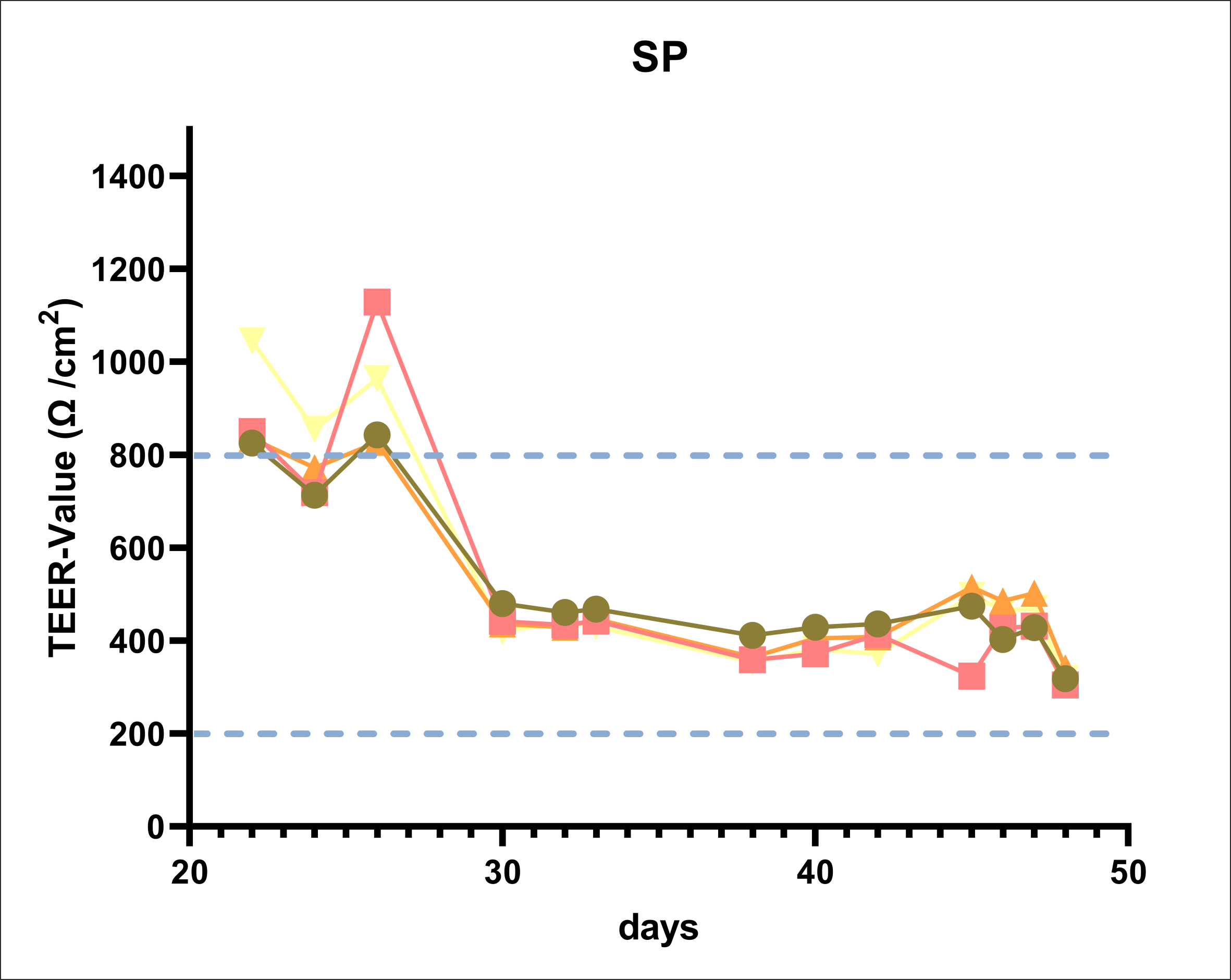

Supplement: Supplementary file 2 — Supplementary Material 2: Supplementary Fig. 2 Evaluation of the physical epithelial barrier by TEER measurements. Depicted are the TEER measurements of SPALI-culture (n = 1) showing particularly slow differentiation behavior. The TEER measurement of differentiation started on day 21 after induction and the measurement was continued until day 48. Each point represents one measurement of all wells of the respective ALI culture at one timepoint. The ALI culture reveals a decrease starting at day 26 and settles on a constant resistance of 800 − 200 Ω/cm2 from day 30–48 onwards [file 12950_2026_497_MOESM2_ESM.tif]

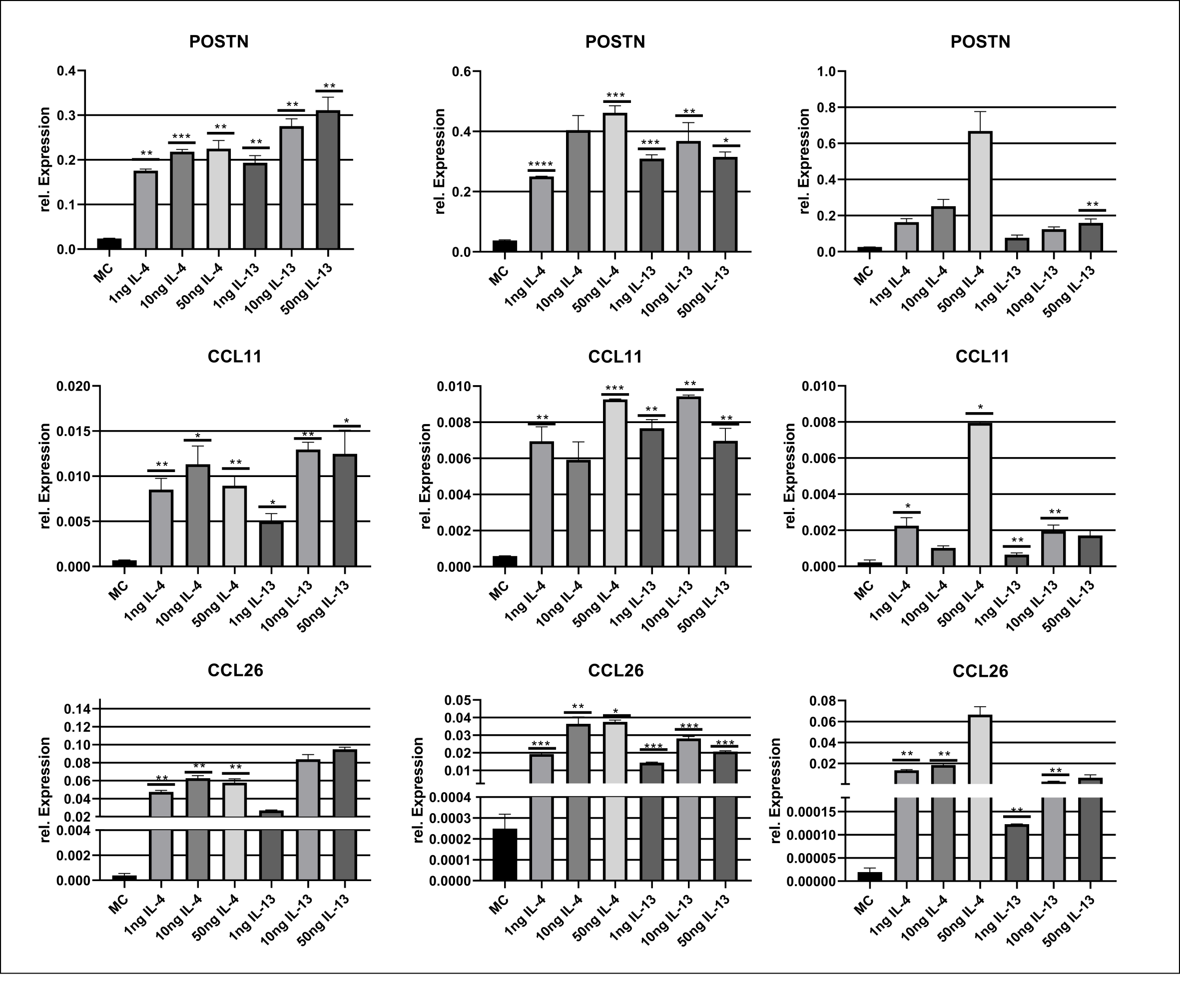

Supplement: Supplementary file 3 — Supplementary Material 3: Supplementary Fig. 3 Stimulation of SPDF (n = 3) with various concentrations of IL-4 and IL-13 (1ng/mL, 10ng/mL and 50ng/mL) to determine the optimal stimulation concentration. Fibroblasts were allowed to grow to confluence, weaned for 24 h under serum-free conditions, and then treated for 24 h. The expression changes of POSTN, CCL11 and CCL26 normalized to GAPDH were examined. For further experiments, a concentration of 1ng/mL was used for IL-4 and 10ng/mL for IL-13 (Welch´s test, two Tailed, 95% confidence interval, ** p < 0.01, *** p < 0.001, ****p < 0.0001 compared to MC) [file 12950_2026_497_MOESM3_ESM.tif]

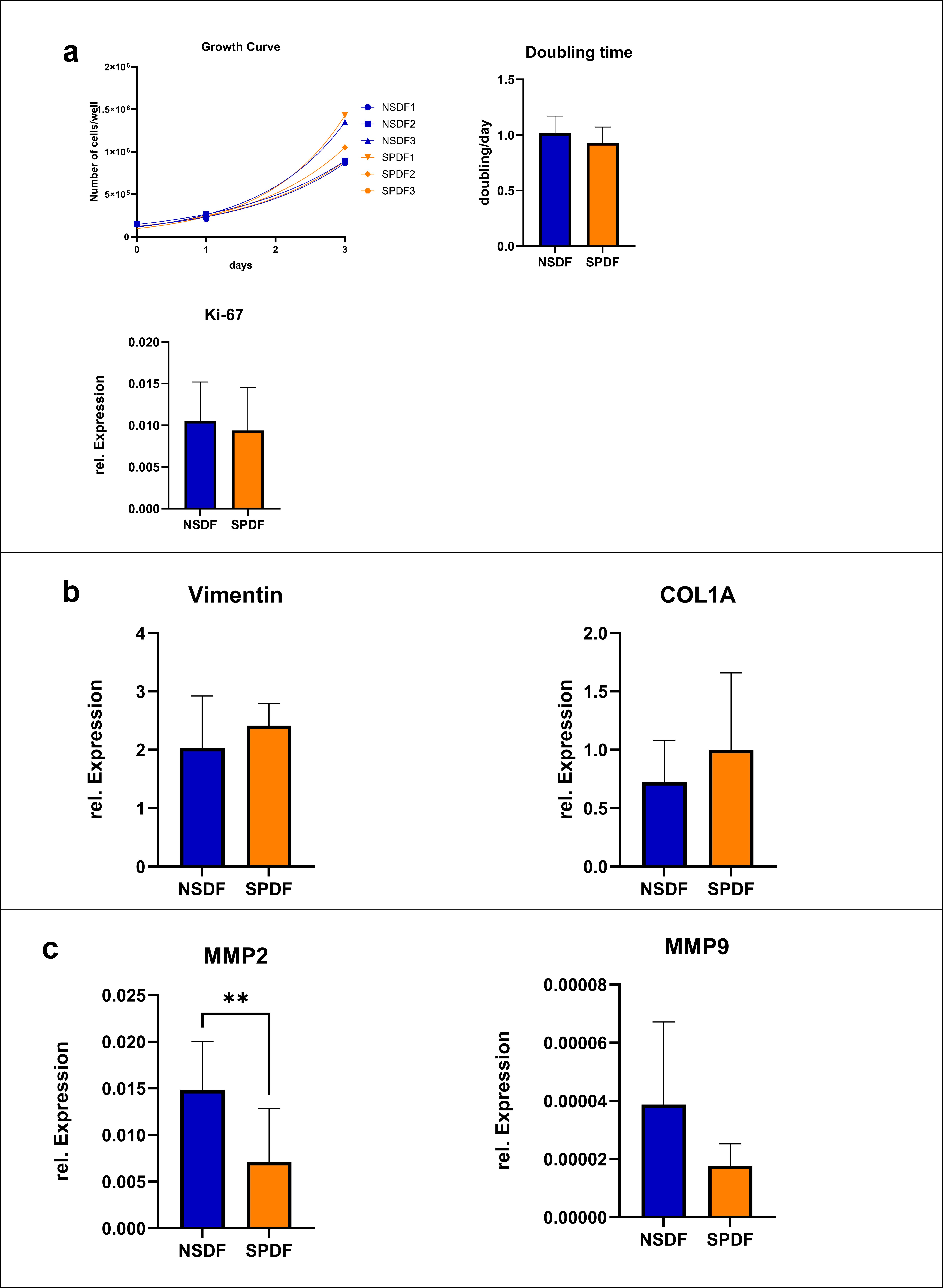

Supplement: Supplementary file 4 — Supplementary Material 4: Supplementary Fig. 4 Evaluation of growth, differentiation and migration-associated genes in NSDF (n = 3) and SPDF (n = 3). (A) Growth experiments of NSDF and SPDF) on average demonstrated no statistically significant differences in cell division (p = 0.513) or proliferation regarding Ki-67 expression (p = 0.63). (B) Investigations regarding differentiation status revealed no significant differences in the expression of vimentin (p = 0.258) and COL1A (p = 0.29). (C) Analysis MMPs expression revealed that NSDFs have significantly higher baseline expression on average of MMP2 compared to SPDFs (p = 0.008). However, MMP9 did not exhibited statistically higher expression in NSDF (p = 0.076). All expressions were normalized to GAPDH (Welch´s test, two Tailed, 95% confidence interval, ** p < 0.01) [file 12950_2026_497_MOESM4_ESM.jpg]

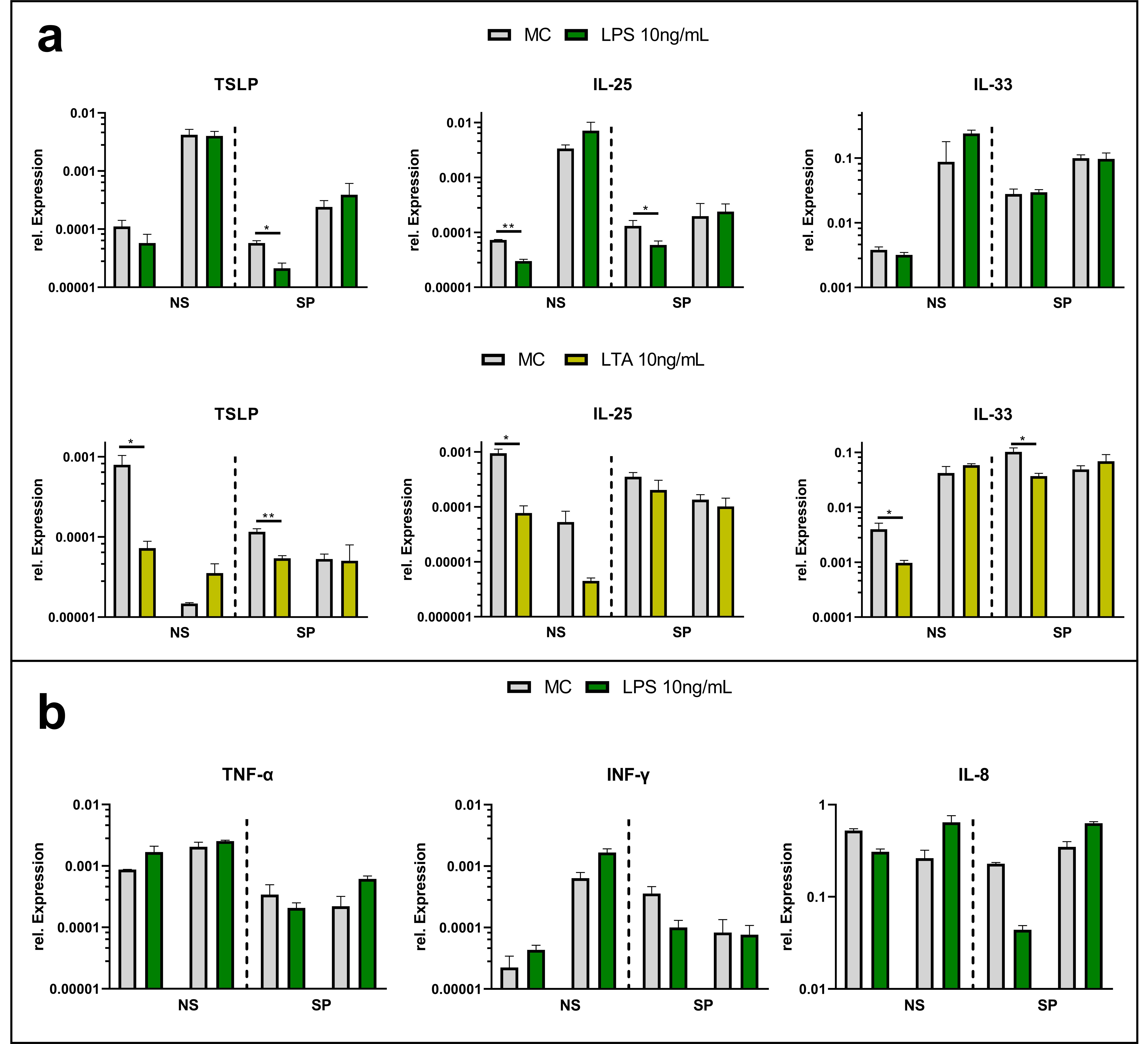

Supplement: Supplementary file 5 — Supplementary Material 5: Supplementary Fig. 5 Expression of key Th1 and Th2 mediators in ALI cultures derived from the respiratory epithelium of CRSsNP (n = 2) and CRSwNP (n = 2), normalized to GAPDH. (a) Neither LPS (TLR4 agonist) nor LTA (TLR2-agonist) were able to induce a consistent upregulation of the TSLP, IL-25 and IL-33 genes. In contrast a partial downregulation of these genes was observed in both cell types, NSDF and SPDF. (b) Treatments with 10ng/mL LPS resulted in no reproducible upregulation of either TNF-alpha and INF-gamma or IL-8 in CRSsNP and CRSwNP derived ALI cultures. (Welch’s test, two Tailed, 95% confidence interval, * p < 0.05, ** p < 0.01) [file 12950_2026_497_MOESM5_ESM.jpg]

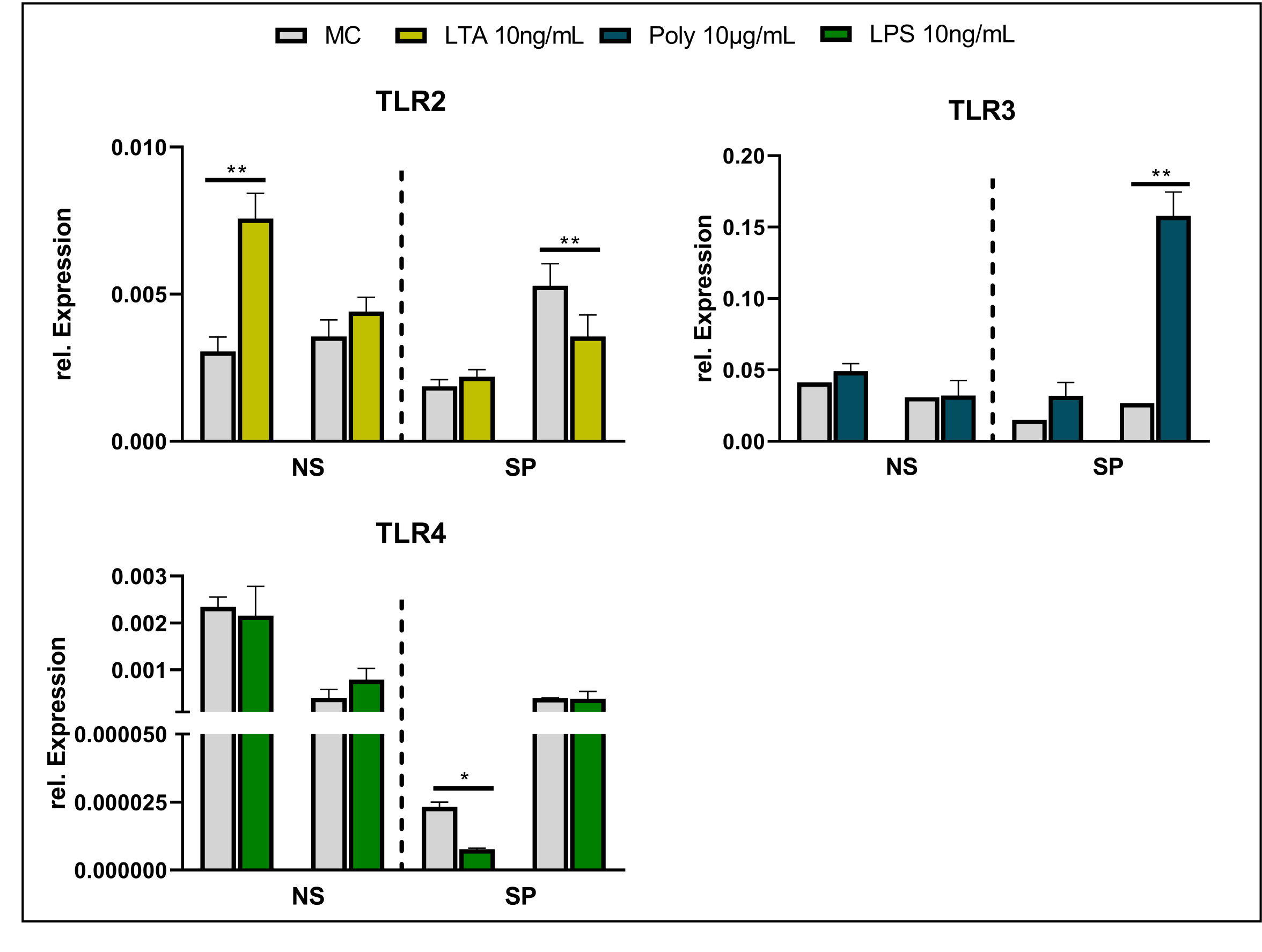

Supplement: Supplementary file 6 — Supplementary Material 6: Supplementary Fig. 6 The expression of TLRs from NS (n = 2) and SP (n = 2) derived ALI cultures of nasal epithelial cells, normalized to GAPDH. The cells were treated with 10ng/mL LTA (TLR2 agonist), Poly(I: C) (TLR3 agonist) or LPS (TLR4 agonist) for 24 h and the expression of the respective receptors was analyzed. In NS- and SP-ALI, neither LTA nor LPS are able to upregulate their respective TLR, whereas Poly(I: C) is capable of upregulating TLR3 in SP-ALI. (Welch’s test, two Tailed, 95% confidence interval, * p < 0.05, ** p < 0.01) [file 12950_2026_497_MOESM6_ESM.tif]

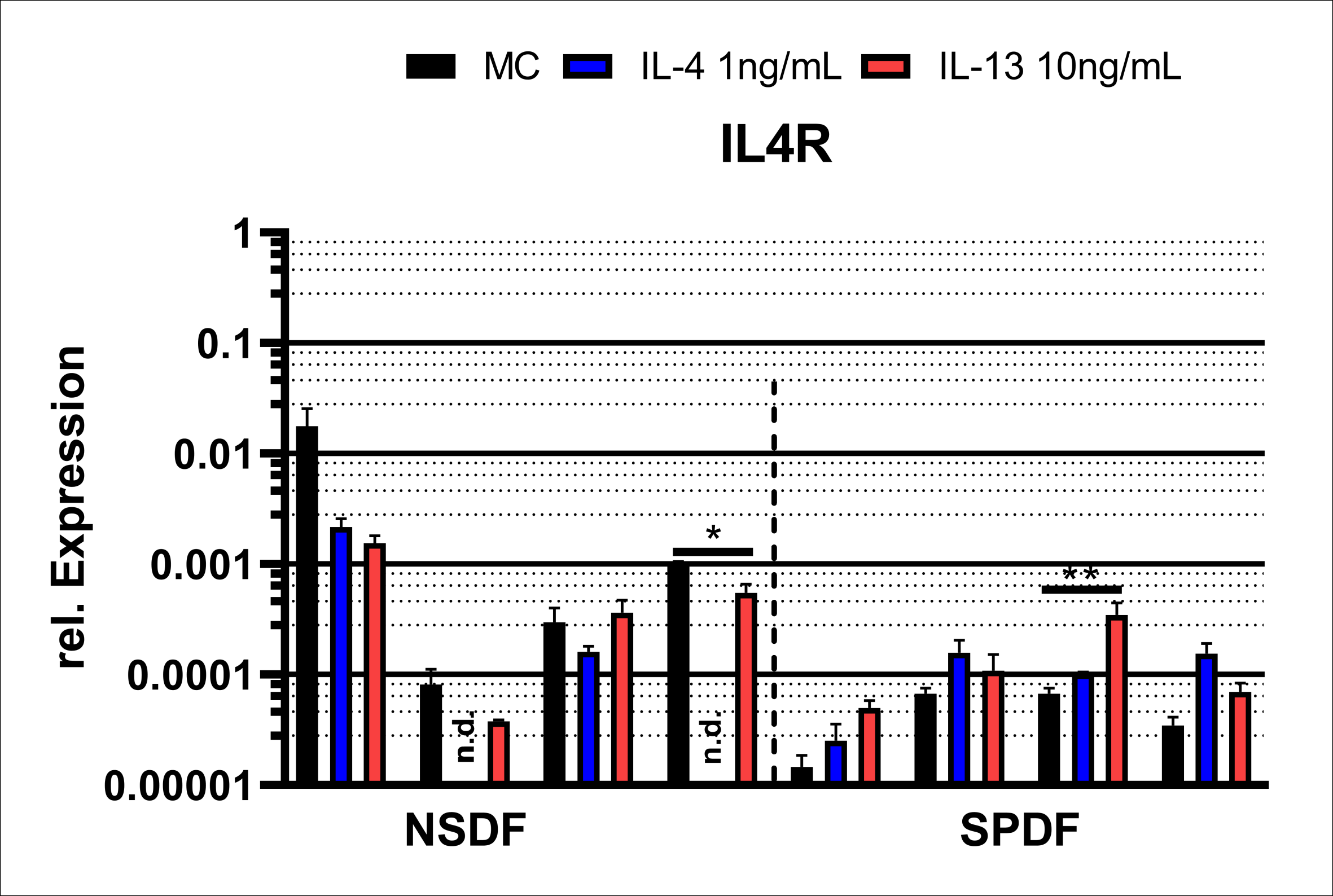

Supplement: Supplementary file 7 — Supplementary Material 7: Supplementary Fig. 7 Investigation of IL4R expression of NSDF (n = 4) and SPDF (n = 4) by treatment with IL-4 and IL-13. Overall, SPDF exhibited increased and more consistent expression of this receptor subunit, while no increases was observed in NSDF (Welch’s test, two Tailed, 95% confidence interval, * p < 0.05, ** p < 0.01) [file 12950_2026_497_MOESM7_ESM.tif]
